# Editorial Independence for *EHP*

**DOI:** 10.1289/ehp.12927

**Published:** 2009-07

**Authors:** Linda S. Birnbaum, Hugh A. Tilson

**Affiliations:** Director, NIEHS, Research Triangle Park, North Carolina, E-mail: birnbaumls@niehs.nih.gov; Editor-in-Chief, EHP, E-mail: tilsonha@niehs.nih.gov

Over the years, *Environmental Health Perspectives* (*EHP)* has emerged as one of the leading journals in the environmental health sciences. It can be argued that one reason for *EHP*’s standing in the field of environmental health is its credibility as an unbiased source of news and research. With the National Institute of Environmental Health Sciences (NIEHS)—the journal’s parent agency—under new direction, we felt it was important to assure our readership that we greatly value the journal’s trustworthy reputation and that we are committed to maintaining its credibility as a high-quality, independent, peer-reviewed publication.

The mission of *EHP* is to serve as a forum for the discussion of the interrelationships between the environment and human health by publishing in a balanced and objective manner the best peer-reviewed research and the most current and credible news in the field. *EHP* does this by maintaining a strict separation from advocacy and nonprofit groups, industry, and government agencies, including the NIEHS.

In reference to the ability of the journal to report scientific findings independent of influence from some segments of the industrial sector, U.S. Representative Dennis Kucinich remarked in a congressional hearing (House Oversight and Government Reform Subcommittee on Domestic Policy 2007) that “*EHP* alone is a pillar of truth.”

In June 2007, a roundtable meeting (Listening Session and Roundtable Discussion on *Environmental Health Perspectives*) was held in Bethesda, Maryland, to consider the future of the journal. Participants included representatives from the environmental health sciences, journal subscribers, academics, and publishers. As part of the discussion, the participants were asked to identify threats that could weaken the journal in the future. One of these threats was the potential for the director of the NIEHS to influence, or be perceived to influence, the journal content. Because *EHP* receives considerable support for personnel and publication costs from the institute and because the journal and its staff are subject to the same organizational rules and regulations as other federal employees, it is logical to be concerned that the NIEHS director might exercise control over journal operations and publication decisions.

The roundtable participants further noted that the potential problem for *EHP* is not so much that of biased editorializing, but of editorial self-censorship—that is, trying not to offend the powers that be, especially among staff who have been long-term government employees. In the minds of the participants, editors working within a government context may be less likely to act and write provocatively, even though it is the role of editorials, news, and research papers to provoke thought and stimulate discussion.

In fact, the NIEHS director makes no attempt to sway editors or writers, or to influence the peer-review process. In our view, editorial independence is an absolute necessity for producing a successful scientific journal. An *EHP* editorial published in January 2008 noted that the content, scope, and direction of the journal would not be influenced by NIEHS leadership and that the editor-in-chief would be given full responsibility for directing and managing all aspects of the publication process (Tilson 2008). Accordingly, editorial decisions about which papers are accepted or rejected, the layout of the publication, and content of the news and editorials would not be subject to approval by NIEHS management. This policy still stands.

We acknowledge and believe it is in the best interest of the NIEHS to support a prominent journal dedicated to stimulating new ideas and publishing research that sustains and develops themes relevant to the mission of the institute but that is not influenced by the institute. It is in the best interest of the institute to ensure that the message of the journal is viewed as fair and balanced to all sides of a question and that the journal is dedicated to publishing the best peer-review work in the discipline of environmental health sciences.

At issue are the credibility and scientific integrity of both *EHP* and the NIEHS. A high degree of credibility and scientific integrity is necessary to maintain the trust of Congress and the public, which are the ultimate consumers and supporters of environmental health science research.

## Figures and Tables

**Figure:**
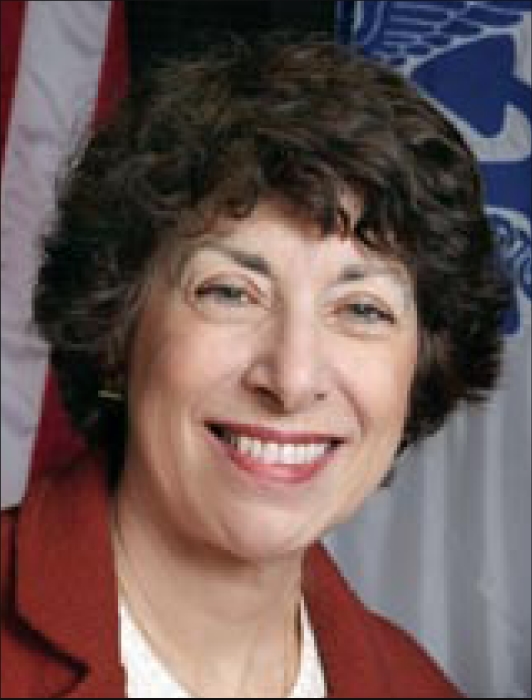
Linda S. Birnbaum

**Figure:**
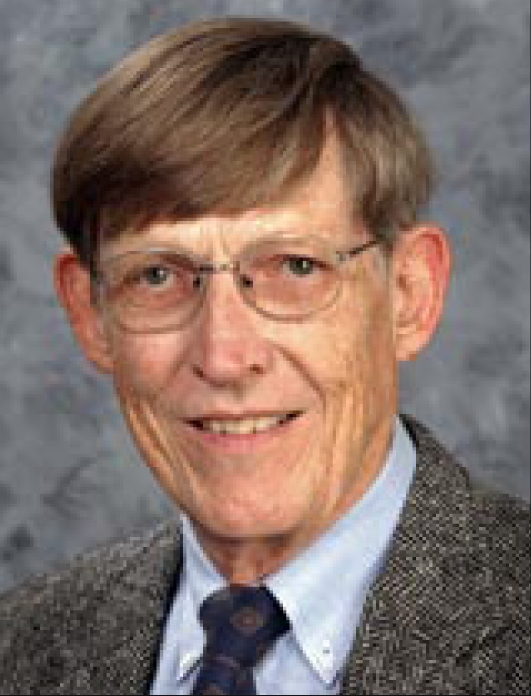
Hugh A. Tilson
